# Aging Mice Show a Decreasing Correlation of Gene Expression within Genetic Modules

**DOI:** 10.1371/journal.pgen.1000776

**Published:** 2009-12-18

**Authors:** Lucinda K. Southworth, Art B. Owen, Stuart K. Kim

**Affiliations:** 1 Biomedical Informatics, Stanford University, Stanford, California, United States of America; 2 Statistics, Stanford University, Stanford, California, United States of America; 3 Developmental Biology, Stanford University, Stanford, California, United States of America; The University of North Carolina at Chapel Hill, United States of America

## Abstract

In this work we present a method for the differential analysis of gene co-expression networks and apply this method to look for large-scale transcriptional changes in aging. We derived synonymous gene co-expression networks from AGEMAP expression data for 16-month-old and 24-month-old mice. We identified a number of functional gene groups that change co-expression with age. Among these changing groups we found a trend towards declining correlation with age. In particular, we identified a modular (as opposed to uniform) decline in general correlation with age. We identified potential transcriptional mechanisms that may aid in modular correlation decline. We found that computationally identified targets of the NF-*Κ*B transcription factor decrease expression correlation with age. Finally, we found that genes that are prone to declining co-expression tend to be co-located on the chromosome. Our results conclude that there is a modular decline in co-expression with age in mice. They also indicate that factors relating to both chromosome domains and specific transcription factors may contribute to the decline.

## Author Summary

There is mounting evidence that mammalian aging is marked by increased gene transcriptional variation. This trend was shown not only by studying gene expression in single cells (Bahar et al. 2006), but at the coarse tissue resolution as well (Somel et al. 2006; Li et al. 2009). These led us to believe that looking at absolute changes in expression level alone may not tell the whole story of transcriptional changes in age. Instead the story may be in the more subtle changes in the coordination of expression among multiple genes. For this reason, we decided to look at changes in co-expression relationships with age. To this end, we developed a methodology for differential co-expression network analysis for the comparison gene co-expression on a global scale. We applied this methodology to compare co-expression between young (16-month) and old (24-month) mice. This allowed us to find both gene groups whose coordination appear to be affected by age and to propose potential mechanisms for the change. We believe our work is of broad importance because it represents a different paradigm for looking not only at aging but also at any complex condition or disease—away from changes in individual genes towards changes in gene relationships.

## Introduction

Since the introduction of DNA microarrays over a decade ago, it has become possible to use genome-wide approaches to explore differences between two biological conditions, such as tumor versus healthy samples, mutant versus wild-type cells or old versus young tissues. The most common type of analysis, called differential expression analysis, looks for genes whose expression changes between two or more different groups. In addition to individual genes, differential expression analysis can also identify groups of genes or pathways that change expression levels in an experiment. For example, pathway analysis shows that genes involved in the electron transport chain show a general decrease in expression with age, even though individual genes in this pathway may not show a large effect [1].

A different type of analysis, differential network analysis, is to create a genome-wide network of genes, and then to look for changes that occur in the network. Gene co-expression networks give insight into how genes work together in particular pathways or systems across multiple microarray conditions. Because most biological processes arise from the complex interactions among multiple gene products, information about how genes function together can improve our understanding of the underlying biological mechanisms. For instance, Hughes et al. used DNA microarrays to profile expression of every gene in yeast in 300 different mutants and chemical treatments, and then calculated which genes were co-expressed with each other under these diverse conditions. This work clearly showed that genes could be grouped into cellular pathways based on co-expression, and provided a useful approach to categorize the function of unknown genes on a global scale [2]. Since this finding, gene co-expression networks have been constructed using worm, fly, mouse, and human microarray data [3]–[6]. In addition, the comparison of co-expression links between orthologous genes in multiple species allows one to search for relationships that are functionally conserved [6],[7].

Looking at how gene co-expression relationships change between two networks is a potentially powerful way to obtain a holistic view of how gene co-expression relationships change between two states. However, searching for differences in networks requires great sensitivity to the initial choice of data. For example, the absence of a shared link in mouse and human co-expression networks does not necessarily indicate divergent function. Instead, differences in the mouse and human co-expression networks may indicate differences in the technical platforms or the experimental conditions used to build the networks.

In this work, we present a novel method for differential co-expression network analysis. Past research has focused on differences in co-expression between networks. Ihmels et al. developed the differential clustering algorithm (DCA) to identify groups of co-expressed genes that differ between yeast species [8]. Choi et al. created a nonweighted co-expression network using a collection of published cancer arrays and compared it to a network composed of the control arrays [9]. Here, we describe a comprehensive and scalable methodology for differential co-expression network analysis and apply it to search for differences in gene co-expression networks during aging in mice.

**Figure 1:**
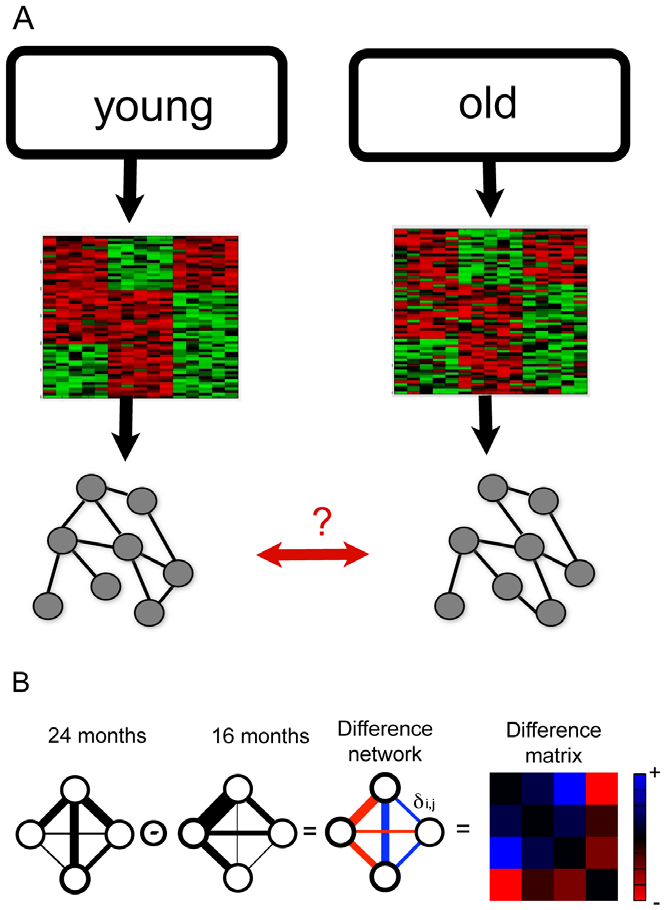
A novel approach for differential co-expression network analysis. (A) Collections of microarrays can be used to look for differences in co-expression networks with age. (B) Each edge in the difference network represents the change in correlation that occurs between young and old mice. The edge weights in the 16-month and the 24-month networks are a function of the correlation in expression between two genes. The edge weights in the difference network represent the magnitude of the difference in correlations. The correlation difference matrix view is a heat map representation of the difference network. In both the network view and the matrix view, red represents a decrease in correlation, and blue represents an increase.

Aging affects a myriad of genetic, biochemical and metabolic processes, and thus it is attractive to use a network approach to globally characterize changes in old age. Genes do not work alone, but rather act within a functional group or pathway, such as a metabolic pathway or a regulatory network. One effect of aging may be to diminish the coherence in expression of gene pathways. In old animals, some genes in a pathway may not be fully activated in tissues that require the function of the pathway, and other pathway components may not be fully repressed in tissues in which the function of the pathway is not needed. In this case, old mice would show less correlation in expression for genes in the pathway than young mice. We test this possible effect of aging by comparing co-expression networks between young and old mice (Figure 1A).

## Results

We used a co-expression network approach to find differences in mice during aging. We first generated separate gene co-expression networks for young and old mice. We used the data from AGEMAP, a large DNA microarray study of gene expression as a function of age [10]. Specifically, AGEMAP studied gene co-expression in C57BL6 mice aged 16 months and 24 months. For each age group, the study examined expression in 16 different tissues. The array platform is a spotted radioactive array with over 12,000 unique cDNA clones stemming from gonad, ovary, and pre- and postimplantation embryos [10]. We discarded the data from three tissues (liver, bone marrow, and striatum) due to missing or poor-quality arrays. For each of the remaining 13 tissues, there are five male and five female biological repeats, resulting in a total of 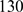 arrays. Each gene array contains 12,273 cDNA clones from the mouse genome. Of these, we discarded expression data from 3,169 cDNAs due to low overall expression (see Materials and Methods).

Instead of looking for genes that change expression with age, we analyzed changes in how genes are linked together by co-expression. Using the expression data that passed the quality controls described above, we generated a gene expression network for each age. For each age group we calculated the Spearman correlation across the 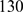 arrays for every pair of genes. In each subset of genes from the network, the few gene pairs that show high correlation are more relevant than the large number of gene pairs that show little or no correlation. We also transformed the Spearman correlation 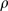 by Fisher's transformation 
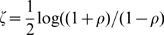
, to get better resolution for the largest most biologically interesting correlations.

We created weighted co-expression networks for each age group in which the nodes in the network correspond to genes and the edges are the non-negative Fisher transformation of the Spearman correlation in expression between two genes. One approach to comparing the young and old gene networks is to separately count the number of edges in each network. With an edge threshold of 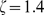 (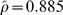), we found that there are 26% fewer total edges (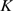) in the 24-month-old network than in the 16-month-old network. In both networks, the connectivity of each gene in the network follows a power law distribution: 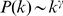, where 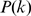 is the probability that a node in the network is connected to 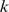 other nodes. For the young network, we estimate 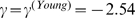, and for the old network, we estimate 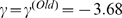.

For the measurements of 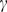 and 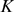, we used a permutation test to ascertain the significance of the observed differences in values between the young and old networks. In the permutation test, the age labels of the mice are permuted, and the data are reconstructed using the new labeling (see Materials and Methods). Significance is determined by the fraction of the permuted test statistic values that exceeds these observed test statistic. The permutation approach eliminates artifacts arising from bias in the data that are not due to aging itself, such as a small number of sick mice or bad gene array experiments that appear in one age group but not in the other. In 1000 permutations the permuted difference 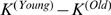 exceeded the observed value 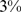 of the time. This gives a one-tailed 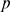 value of 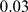. To be cautious we report a two-tailed 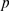-value of 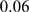. The observed value of 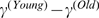 exceeded the permuted values in 24% of the cases (two tailed 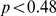). Thus the connectivity slope does not differ significantly between the groups, while the total number of edges has at most borderline significance.

### Difference networks identify groups of genes that change co-expression with age

We developed a difference network framework to directly identify groups of genes that change correlation with age. In the difference network framework, every node represents a gene, and every weighted edge represents the change in correlation between old and young mice for the corresponding gene pair (Figure 1B). The edge weights, 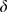, are scalars such that a negative 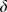 represents a decrease in correlation and a positive 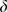 represents an increase in correlation:
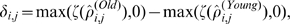
(1)where 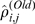 and 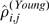 are the Spearman correlation coefficients between genes 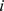 and 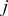 in the 24 month and 16 month data sets respectively, and 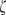 is the Fisher transformation. The Fisher transformation, when applied to a sample correlation coefficient 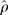, yields an approximately normally distributed estimator. Though changes in negative correlations could also be of interest, this choice of 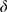 allows us to focus on changes in positive correlations.

The most straightforward way to evaluate the difference network is to determine whether the average is positive or negative. However, in the difference network, there are a total of approximately 36 million weighted edges, a vast majority of which involve pairs of genes that are not co-expressed and thus have values that are near zero. The small fraction of edges that reflect true co-expression difference would likely be obscured by the large number of edges that do not change with age.

We reduced the complexity of the difference network problem by focusing on groups of genes rather than individual gene pairs. We used two different classification methods to define gene groups: *(a)* genes that show strong co-expression in a related set of gene array experiments and *(b)* genes that are within the same function group as defined by the Gene Ontology (GO) classification.

**Figure 2:**
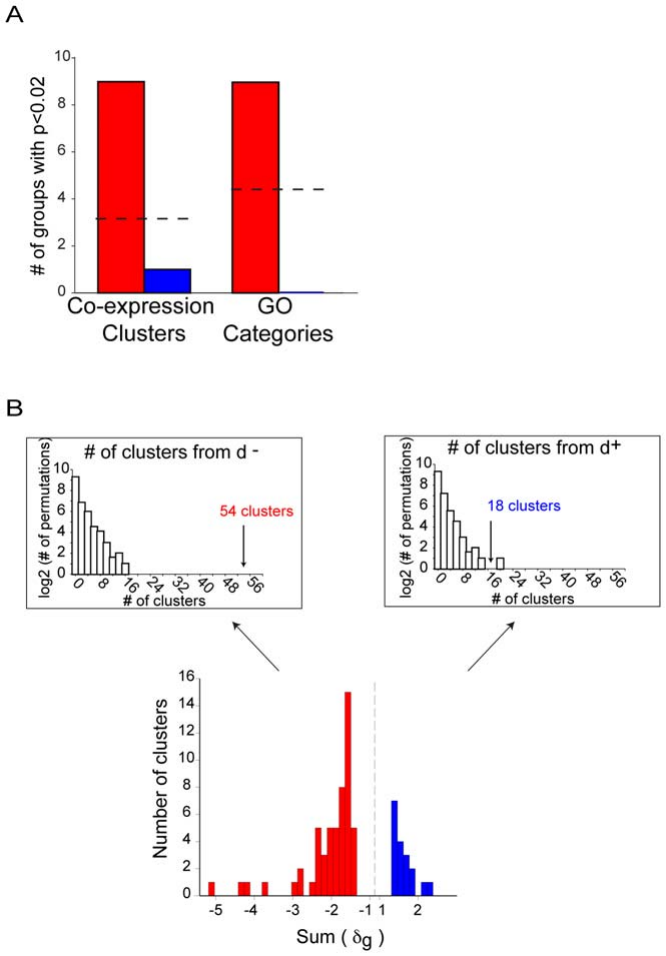
Functional gene clusters tend to decrease with age. (A) The number of co-expression- and Gene Ontology (GO)- defined groups that change with age. The red bars indicate decreasing co-expression, and the blue bars indicate increasing co-expression. The dotted line represents the number of groups expected under the null distribution. (B) Histogram of the sum of the edge weights for the clusters using 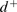 and 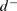. Again the red bars indicate decreasing co-expression, and the blue bars indicate increasing co-expression. Top left panel: The total number of groups that decrease in co-expression exceeds any of the 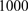 permuted values. Top right panel: The total number of groups that decrease in co-expression exceeds all but 4/1,000 of the permuted values.

The first classification method involved finding groups of genes that are co-expressed. We could not use the gene array data from either the 16- or the 24-month-old mice because this would bias the analysis. For example, a gene set defined by co-expression in the 16-month data set naturally has higher co-expression than it would in the 24-month data set, and vice versa. For this reason, we used a separate gene expression data set, the AGEMAP compilation of data from 6-month-old mice. Like the 16- and 24-month AGEMAP data, the 6-month data included 10 gene arrays from 13 different tissues. Using the gene expression data from this set of 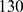 gene arrays, we calculated the correlation in expression between all pairs of genes, then used average-linkage hierarchical clustering to group the genes into clusters (see Materials and Methods). For this analysis, we defined gene clusters using a distance cutoff such that all resulting clusters had an average within-cluster Spearman correlation of at least 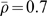. The hierarchical clustering established a list of 312 gene sets containing a minimum of five genes. For each group (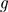), we defined the test statistic (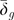) as the mean of the intergroup edge weights in the difference network. For each gene group, we used a permutation test to count how many times the permuted test statistic exceeded the observed 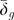. We considered a gene group significantly decreasing if the sample value was below the first percentile of permutations (two-sided 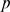-value 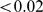). We considered a gene group significantly increasing if the sample value was above the 99th percentile of permutations. From the 312 gene clusters, we found nine clusters of genes that show decreasing correlation with age and one cluster that shows increasing correlation with age (Table 1). Because we tested 312 clusters, we would expect to identify 3.12 clusters of each type (increasing and decreasing) under the null hypothesis (Figure 2A).

**Table 1:** Co-expression clusters and Gene Ontology (GO) categories that change correlation with age at the top 1 percentile.

Group name	%^a^	Description^b^
cluster175	0.0	IPR000225 Armadillo ( 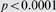 c)
GO:0007613	0.0	Memory
cluster78	0.2	GO:000716 G-protein coupled receptor protein signaling ( 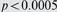 )
GO:0008146	0.2	Sulfotransferase activity
cluster73	0.3	IPR006630 RNA-binding protein Lupus La ( 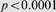 )
		GO:0008173 RNA methyl-transferase activity ( 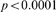 )
		IPR014729 Rossmann-like sandwich fold ( 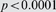 )
		GO:0006898 Receptor-mediated endocytosis ( 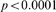 )
cluster306	0.3	GO:0019203 Carbohydrate phosphatase activity ( 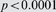 )
		GO:0006118 Electron transport ( 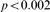 )
GO:0016050	0.5	Vesicle organization and biogenesis
GO:0008430	0.7	Selenium binding
GO:0006940	0.8	Regulation of smooth muscle contraction
GO:0006518	0.8	Peptide metabolic process
cluster120	0.9	-
cluster68	0.9	GO:0000079 Cyclin-dependent protein kinase activity ( 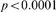 )
		KEGG:00190 Oxidative phosphorylation ( 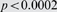 )
GO:0005048	0.9	Signal sequence binding
cluster201	0.9	GO:0015935 Small ribosomal subunit ( 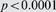 )
cluster17	0.9	-
cluster178	0.9	GO:0005925 Focal adhesion ( 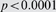 )
GO:0030057	0.9	Desmosome
GO:0031253	0.9	Cell projection membrane
cluster14 	99.50	KEGG:00350 Tyrosine metabolism ( 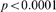 )
		KEGG:04514 Cell adhesion molecules (CAMs) ( 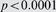 )

a The percentile is calculated as the percent of permutation that exceeds the true correlation difference. The percentile corresponds to 100×[one-tailed p-value. Gene groups marked with a * indicate that the correlation is increasing with age.

b If a co-expression cluster is enriched for genes in a GO category, genes in a KEGG category, or genes that share a protein motif from the Interpro (IPR) database, then the associated category is listed [48],[49].

c The *p*-value for enrichment is calculated using the hypergeometric distribution (see Materials and Methods).

We inspected the expression pattern of the genes for the ten clusters that change co-expression with age, and noticed an interesting pattern regarding expression in the gonads and adrenal glands. The genes from two clusters (clusters 175 and 14) are expressed at high levels specifically in the gonads and adrenal glands, and the genes from four clusters (clusters 78, 73, 68 and 178) are broadly expressed except for low expression in the gonads and the adrenals (Table 1). The gonads and adrenals both produce steroid hormones. The ovaries produce estrogen and progesterone, the testes produce testosterone and the adrenal glands produce corticosteroids such as ACTH. In all cases, steroid production decreases with age.

In addition to grouping genes based on co-expression, we also grouped genes based on shared genetic functions using GO categories. We used gene sets that shared the same GO molecular function, associated cellular component, or biological process. GO categories are useful in that they provide a functional grouping of related genes, and genes with similar functions are often co-expressed.

We used 395 GO categories containing 5 to 200 genes with minimal overlap to test for group-wide correlation changes with age. We identified the set of GO categories by looking at the categories at every level of distinction, and discarded any group that has more than 50% overlap with any other category that is smaller than it. As before, we used permutation to test for significance of within-group edge weight change. We found that nine GO categories decrease correlation with age, and zero categories increase correlation with age (Figure 2A).

Table 1 lists the GO categories that change significantly with age. The top GO category includes genes involved in memory, which clearly declines with age. Another interesting GO category is selenium binding. Selenium is a trace element that acts as a cofactor for reduction of antioxidant enzymes [11]. Several studies have suggested that low levels of selenium may be a risk factor for developing cancer in humans [12]. Changes in the genes responsible for selenium binding with age would have interesting implications for the role of antioxidants in aging.

### Identifying dense areas of change in the aging difference network

The observation that gene sets tend to show an overall decline in correlation of gene expression with age suggests that there may be densely-connected subgraphs of negative edges in the gene-correlation difference network. To find areas of the difference network that loosen with age, we clustered genes using the weighted edges of the difference network as a distance matrix. For example, to find a densely-connected subgraph of the difference network where all of the edges are negative (i.e., decreasing with age), we define the distance metric between two genes 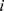 and 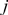 to be 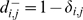. Thus, a gene pair that decreases correlation with age has a small clustering distance. If we cluster using the distance matrix composed of 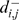 for 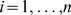 and 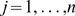, then the resulting clusters of genes are chosen based on their shared correlation loss. Similarly, if we set the distance measurement to be 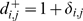, then two genes that increase correlation with age are separated by only a small distance. Clustering via 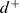 will yield clusters in which the members increase correlation with one another.

We clustered the genes in the difference network using 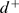 and 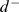 to locate clusters that increase and decrease respectively. We used average linkage hierarchical clustering with a set distance cutoff of 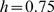 to define clusters. We set the height cutoff to 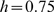 so that all of the resulting clusters had a mean average correlation difference of 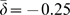 or 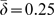 for 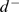 and 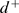, respectively. Using a minimum size cutoff of five probes, we found 54 clusters that decreased correlation with age and 18 clusters that increased correlation with age (Figure 2B).

As shown in Figure 2B, by using permutations to test for significance, we found that the number of clusters identified via 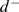 and 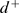 are both significant. For 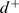 the true number of clusters fell in the top 0.4 percentile (corresponding to two-tailed 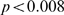). For 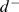 the true number of clusters far exceeded any of the permuted values. Although both clusterings are significant, there are more clusters that decrease in correlation than increase in correlation with age (Figure S1).

From the difference network clusters obtained using 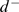 and 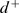, we located interesting groups of genes that change correlation with age. Table 2 and Table 3 describe the clusters we found using 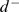 and 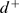, respectively. Among the clusters that decreased correlation with age are many gene groups previously implicated in aging pathways, such as mitochondrial function, transcriptional regulation, and ribosome biogenesis. One cluster, enriched for DNA-damage genes, shows increasing correlation with age. Because DNA damage increases with age [13], it is possible that DNA-damage pathways are more frequently triggered in old age, producing a more coordinated transcriptional response.

**Table 2:** Groups of genes defined using the cluster distance 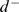.

Cluster^a^	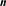	GO ID^b^	GO description	Enrichment^c^
cluster 5	8	GO:0031966	mitochondrial membrane	0.0004
cluster 7	8	GO:0003779	actin binding	0.0001
cluster 8	5	GO:0005344	oxygen transporter activity	0.0001
cluster 10	6	GO:0006888	ER to Golgi vesicle-mediated transport	0.0001
cluster 16	6	GO:0022613	ribonucleoprotein complex biogenesis and assembly	0.0002
cluster 21	5	GO:0006461	protein complex assembly	0.0001
cluster 24	8	GO:0006260	DNA replication	0.0002
cluster 25	6	GO:0043066	negative regulation of apoptosis	0.0001
cluster 27	7	GO:0048568	embryonic organ development	0.0001
cluster 28	5	GO:0019318	hexose metabolic process	0.0001
cluster 29	7	GO:0016458	gene silencing	0.0001
cluster 30	17	GO:0016481	negative regulation of transcription	0.0002
cluster 33	7	GO:0009966	regulation of signal transduction	0.0003
cluster 36	6	GO:0008757	S-adenosyl methionine-dependent methyl transferase activity	0.0001
cluster 42	5	GO:0005746	mitochondrial respiratory chain	0.0001
cluster 43	6	GO:0006118	electron transport	0.0001
cluster 48	5	GO:0005624	membrane fraction	0.0001
cluster 49	5	GO:0015630	microtubule cytoskeleton	0.0001
cluster 50	5	GO:0006898	receptor-mediated endocytosis	0.0001
cluster 53	6	GO:0005624	membrane fraction	0.0002
cluster 54	5	GO:0050877	neurological system process	0.0001

a Only clusters significantly enriched for a Gene Ontology (GO) category are listed.

b The GO category with the highest enrichment in the gene group.

c The *p*-value for enrichment is calculated using the hypergeometric distribution.

**Table 3:** Groups of genes defined using the cluster distance 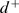.

Cluster^a^	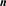	GO ID^b^	GO description	Enrichment^c^
cluster 1	5	GO:0016887	ATPase activity	0.0001
cluster 3	5	GO:0016887	ATPase activity	0.0001
cluster 9	6	GO:0005506	iron ion binding	0.0001
cluster 12	8	GO:0008380	RNA splicing	0.0003
cluster 13	5	GO:0006974	response to DNA damage stimulus	0.0001

a Only clusters significantly enriched for a Gene Ontology (GO) category are listed.

b The GO category with the highest enrichment in the gene group.

c The *p*-value for enrichment is calculated using the hypergeometric distribution.

### Modular loosening of the correlation difference network

One possibility is that there may be a uniform loosening of gene edges throughout the difference network. For example, there may be age-related damage to basic components of gene expression that are used for all genes, such as RNA polymerase II, or there may be damage to chromatin-modification enzymes. If such proteins become damaged in old age, expression of all genes may be affected and may result in decreased levels of gene correlation.

Another possibility is that certain areas of the correlation difference network may show much greater loss of edge strength than other areas. This difference would appear modular, with entire groups of genes losing correlation relative to one another. For example, aging may affect a specific DNA-binding transcription factor, in which case the downstream target genes of the transcription factor would show large age-related losses in gene correlation.

We investigated whether there was uniform or modular loosening of the correlation network with age using two parameters in the unweighted gene co-expression networks: the connectivity and the clustering coefficient. In an unweighted network, the connectivity is defined as the number of neighbors of a given gene. The clustering coefficient measures the degree to which genes cluster together (Materials and Methods). The clustering coefficient ranges from zero (none of a gene's neighbors is connected to any other) to one (all of the neighbors are connected to one another).

**Figure 3:**
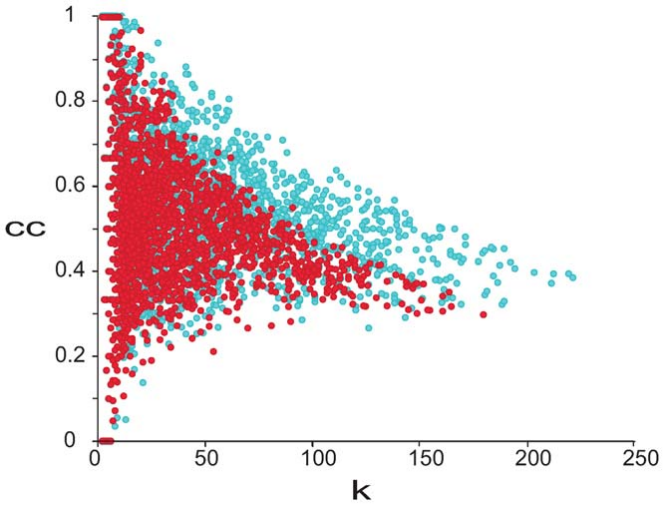
Difference in the clustering coefficient (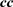) versus the connectivity (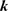) distributions between young and old networks. Each dot represents a probe in either the 16-month-old (*blue*) and 24-month-old (*red*) networks. All of the probes with at least one neighbor are plotted.

Figure 3 shows a plot of the clustering coefficient against the connectivity for the 16- and the 24-month-old gene-correlation networks. We found that there are fewer genes in the upper right side of the plot in the older network than the younger network, implying that large interconnected gene groups tend to be lost as mice age.

**Figure 4:**
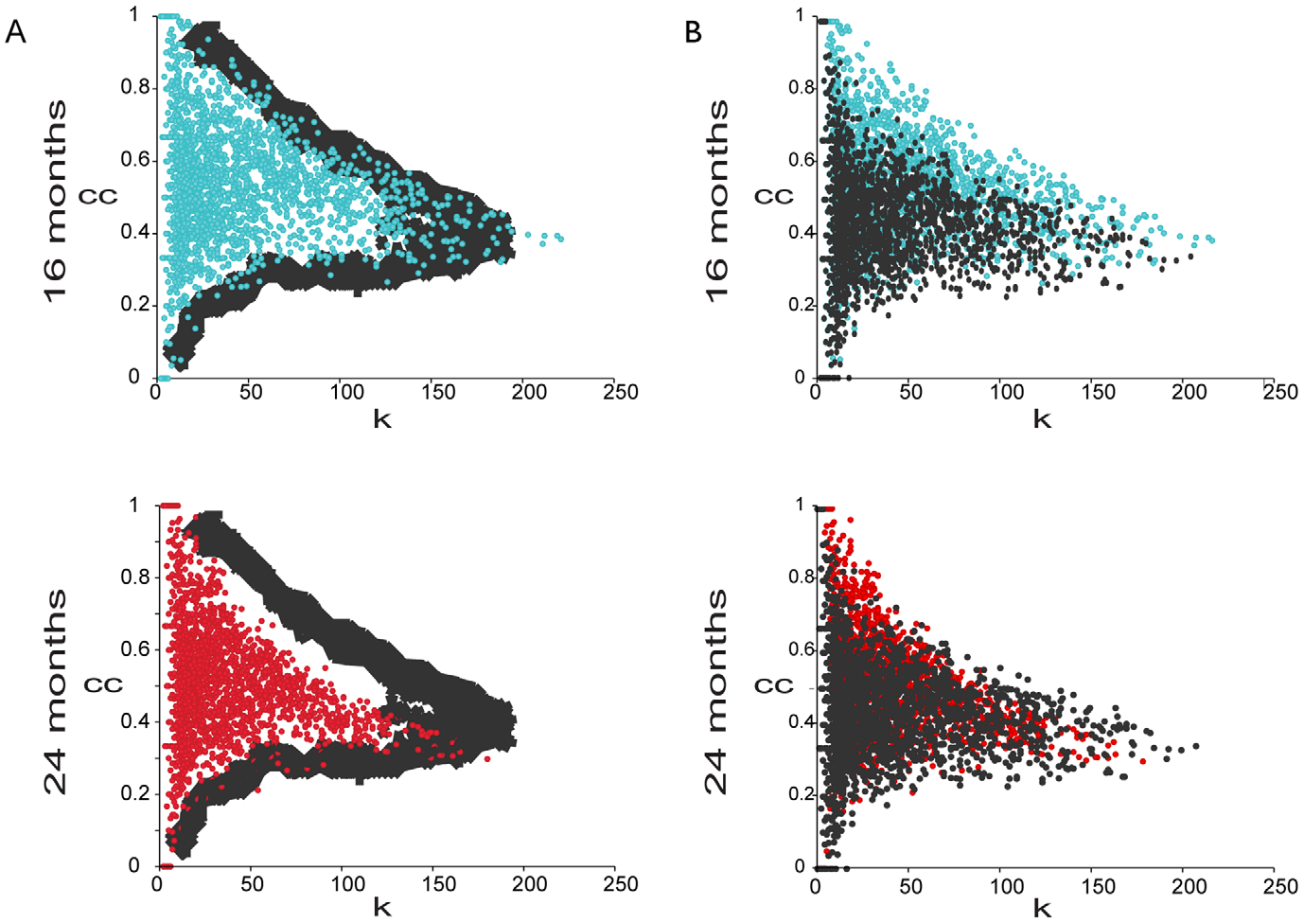
Deletion simulations indicate modular co-expression loss. (A) The clustering coefficient (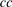) versus the connectivity (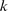) after simulation of uniform co-expression loss. Each dot represents a probe in either the 16-month-old (*blue*) and 24-month-old (*red*) networks. All of the probes with at least one neighbor are plotted. The gray lines represent the 100 node-deletion simulations. (B) The clustering coefficient (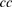) versus the connectivity (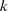) for young and old mice as contrasted with the cluster-based deletion simulation. Each dot represents a probe in either the 16-month-old (*blue*) and 24-month-old (*red*) networks. All of the probes with at least one neighbor are plotted. The distributions from the cluster-based deletion simulations are shown in gray.

We developed simulation tests to determine whether the differences between young and old mice networks could be explained by uniform or modular loosening of gene expression edges. We then compared the observed data to each of the simulations to determine which showed the greatest resemblance. We simulated uniform loosening of the network using a node-based deletion. In the node based simulation we randomly selected nodes in the 16-month-old network and deleted all edges leading out of those nodes. We continued to delete edges until the number of edges in the simulated network was equal to the number of edges in the 24-month-old network. We repeated the simulation 100 times, and each time we drew the boundaries of the simulated networks on the scatter plot of the clustering coefficient versus the connectivity (see Materials and Methods). As shown in Figure 4A, the boundaries of the simulated networks do not resemble the boundary for the 24-month-old network. This result indicates that loss of gene expression correlation in 24-month-old mice does not occur uniformly across the network.

We also modeled networks in which the loss of gene co-epression during aging was modular. We simulated modular correlation loss using a cluster-based deletion, removing co-expressed clusters from the network derived from the young mice. First, we clustered the genes by co-expression in the 16-month-old network. We defined clusters using average linkage hierarchical clustering, with a distance metric of 1-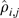, where 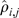 is the Spearman correlation between genes 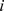 and 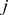. Distinct clusters are formed by cutting the tree at a particular height 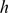 (see Materials and Methods). Next, we deleted all of the clusters from the 16-month-old network using a number of different values of 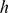. Figure S2 shows the simulation results for various height cutoffs. This figure shows that the correlation loss observed from young to old mice is consistent with removing clusters defined at a 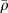 of 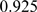 (Figure 4B). These results show that aging gene networks appear to loosen in a modular fashion.

### Transcription factor binding sites

One possible mechanism of modular loosening of a gene expression cluster with age is if all of the genes in a cluster are targets of a specific transcription factor. If a transcription factor loses the ability to co-regulate a group of genes with age, we can expect to see a decline in correlation in expression between those genes as the animals age. To this end, we searched for transcription factors whose targets changed co-expression with each other between young and old mice. Transcription factors contain DNA binding domains that attach to a specific sequence of DNA. The Transfac database lists known sequence motifs to which a transcription factor binds. By identifying all genes that contain a conserved Transfac motif in their upstream regions, we obtain an estimate of genes that may be targets of a particular transcription factor (See Materials and Methods).

**Figure 5:**
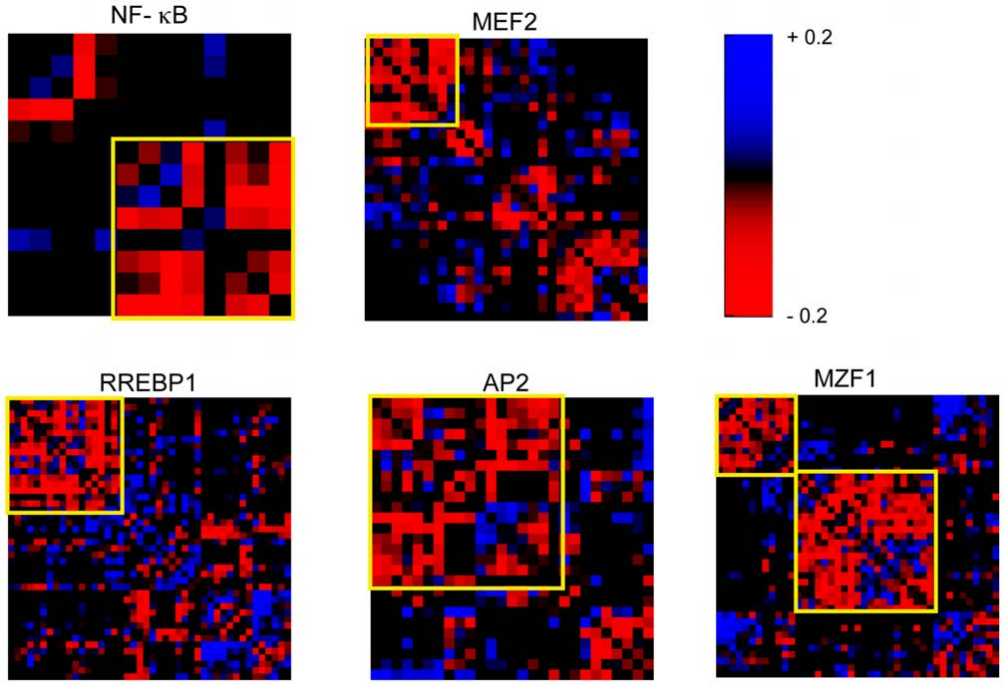
Heat map representation of correlation changes in targets of transcription factors that significantly decrease correlation with age. Red represents a decrease in correlation between two genes, and blue represents an increase. The yellow boxes identify the subsets of genes that are strongly decorrelated.

We downloaded binding information for 258 known conserved transcription factor binding sites from the Transfac database. From all of the transcription factor binding sites, we created 163 gene groups classified according to the presence of a conserved transcription factor binding site within 5000 bp upstream of the translation start site. We only used gene groups that contained five or more unique targets. To each group, we looked at the mean 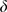 (Equation 1) and assessed significance using the permutation method described in Materials and Methods. We found five transcription factors whose downstream targets decreased correlation with age, where 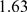 are expected by chance (Table 4, Figure 5). For each of these significant sets of genes, there is a subset of genes that strongly lose correlation in expression relative to one another with age. We did not find any transcription factors whose targets increased correlation in expression with age.

**Table 4:** Transfac-defined gene groups that change correlation with age in the top 1 percentile.

Transcription factor	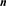 a	%^b^
NF- 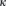 B	13	0.2
MEF2	31	0.4
RREBP1	44	0.6
AP2	27	0.6
MZF1	43	0.9

a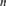 is the number of downstream targets.

b The percentile is calculated as the percent of permutation that exceeds the true correlation difference and corresponds to 100×[one-tailed p-value.

Three of the most significant groups contain genes with binding sites for NF-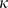
B, AP2, and MEF-2. NF-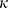
B is involved in cellular inflammation. NF-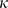
B has a myriad of inducers, such as reactive oxygen species (ROS), infection, and cytokines [14],[15]. All of these factors increase with time and thus have been implicated in aging. The amounts of ROS, a by-product of cellular metabolism, has clearly been shown to increase in old animals. Perhaps because of the increase in ROS, NF-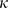
B is abnormally activated in the major lymphoid organs [16].

AP2 is involved in a variety of processes, including morphogenesis and development. Its involvement with aging primarily stems from its regulation of the aging-associated human helicase protein WRN [17]. The targets of MEF-2 also appear to lose correlation in expression with age. MEF2 is a muscle-specific transcription factor that has been shown to increase binding affinity with oxidative stress in human primary skeletal muscle cells [18].

### Chromosomal clustering of genes that lose expression correlation with age

Another mechanism that could account for the unevenness in the correlation loss of the gene co-expression network in old mice is changes in specific chromatin domains. Chromosomes have regions of open chromatin (which are accessible to transcription factors and permit gene expression) and closed chromatin (which does not allow transcription factor binding and prevents gene expression). Domains that are open in young mice may become less open in old mice, and domains that are closed in young mice may become partially open in old mice. If so, genes that are fully expressed from open chromatin domains in young mice may become partially repressed in old mice, and genes that are not expressed in young mice because they are in closed chromatin domains may become partially derepressed in old mice. The net effect of such a loss of regulation could be to show lower correlation levels in old age. In this case, we would expect genes that tend to lose correlation with other genes in old age to be clustered together on the chromosome.

**Figure 6:**
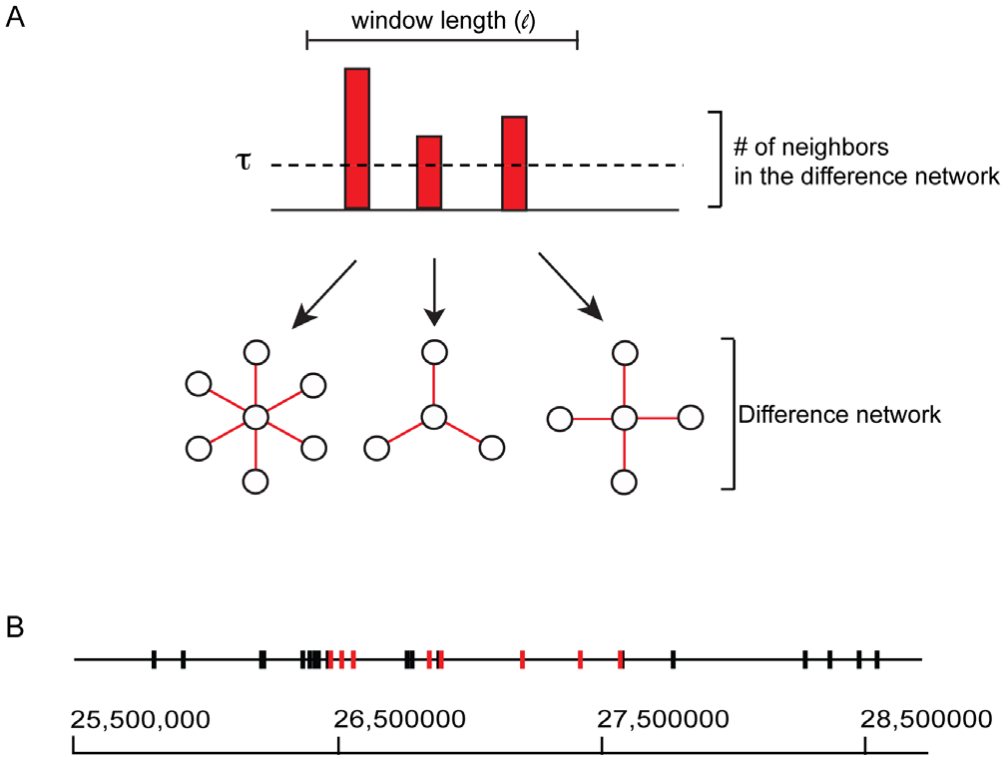
Genes that are prone to correlation loss are clustered on the chromosome. (A) For a window of a set size, genes with connectivity in the difference network above a threshold 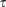 are counted. (B) The positions of genes are plotted on the chromosome. The black bars are genes that do not meet the loss score threshold. Genes with a high loss score (*red bars*) were found to be clustered together.

We tested for a chromatin domain effect by determining whether genes that lose expression correlation with other genes in old age tend to be clustered together or randomly dispersed. We defined neighbors as the genes in the difference network that show a decreasing correlation in expression with the target gene above a set threshold. The number of such neighbors represents an age-related correlation loss score (Figure 6A). Thus a gene that has a high age-related correlation loss score is a gene that loses correlation with many of the genes that it was previously correlated with at 16 months. We scanned the genome with a moving window and counted the number of windows that have two or more genes with a correlation loss score above the threshold. Using a threshold set at six genes and a window size of 80 kb, we identified 44 windows with two or more genes that lose expression correlation with age (Figure 6B).

To determine whether this number is statistically significant, we repeatedly scrambled the locations of the genes and recalculated the number of clusters. The results from 1000 permutations are presented in Figure S3A, which shows that the observed number of windows is greater than the number found by random permutation in all but two cases (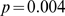). This result indicates that genes that lose correlation in expression with their neighbors in old age tend to be clustered on the chromosome. We repeated this analysis using a number of different thresholds (two to six genes) and window sizes (10 to 200 kb). We found similar results for a range of parameters (Figure S3B), including some even more significant than our original choices. Thus, genes that are sensitive to loss of regulation of expression with age occur in specific regions in the chromosome, perhaps because these regions correspond to chromatin domains that are affected by aging (Table S1).

## Discussion

Here we present a methodology to compare two biological states (young versus old mice) by performing a global comparison of changes in gene co-expression. There is an important difference between comparing changes in expression levels and comparing co-expression relationships. Traditional analysis focuses on finding genes or groups of genes whose expression levels differ between two states. On the other hand, differential co-expression analysis looks for changes in the co-expression relationships between genes. By comparing how the correlation in gene expression differs between two states, we can make inferences about changes in functional interconnectedness of those genes.

Comparing network relationships is not a novel concept, however most such comparisons focus on finding similarities. For example, co-expression networks have been constructed for multiple species by identifying genes that show conserved co-expression with each other among large numbers of DNA microarray experiments [6],[7]. Numerous algorithms have also been proposed to find similarities among different types of biological networks. For example, Walhout et al. combined co-expression data with protein-protein interaction and phenotypic data to obtain information about functional gene interactions in the *Caenorhabditis elegans* germline [19]. Similar approaches that integrate multiple high-throughput data types have been created for various microbes, yeast, worms, and humans [20]–[31].

The above approaches have successfully been used to pinpoint similarities between networks. Searching for differences is a more nuanced problem. In addition to our method, two previous studies have looked at differences in networks [8],[9]. There are several key differences between our algorithm and the previous ones. Our method assigns a statistical significance to the changes in the gene clusters, it uses weighted networks and it allows for the unsupervised identification of changing clusters. Although the previous two algorithms were able to achieve many of these criteria, neither met all of them.

When looking for network similarities, less attention can be given to the composition of the data from which the networks are constructed because similarities in differently constructed networks are likely to be biologically relevant. For example a similarity between an edge in the fly and worm gene co-expression network is likely to indicate a shared functional link between two genes. In contrast, when looking for network differences, more attention needs to be given to the input data so that the comparison shows biological differences rather than artifacts that reflect the manner in which the data were collected. A divergence in that network may be due to a trivial difference in the types of experiments being performed, the experimental platform, the lab that performed the experiment or the experimental design. For example, the differential clustering algorithm (DCA) identifies groups of genes that are co-expressed in one yeast species (*C albicans*) but not another (*S. cerevisiae*), or vice versa [8]. However, the input expression data for the two yeast species are not closely matched. Thus, it is possible that some of the differences observed between *S. cerevisiae* and *C. albicans* arise from a bias in experiment selection rather than intrinsic differences in biological properties.

Bias in the experimental input can be controlled by carefully matching the arrays for each condition. Choi et al. used this method to compare a non-weighted gene co-expression network from cancer samples to a similarly constructed network from normal tissues [9]. By matching each tumor type to the corresponding control from normal tissues, they minimized the potential for experimental bias in the construction of the cancer and normal networks. The AGEMAP data set provides a unique opportunity to create matching networks as the data set from the 16- month old mice is matched to the data set from the 24- month old mice. The mice were raised in the same facility, the data were collected by one lab using one experimental platform and identical experimental protocols. Since the only consistent difference between the 16- and 24-month old data sets is the age of the mice, it is more likely that the differences in gene correlations between the two networks reflect the effects of aging.

We chose to use a network approach to compare young versus old mice because aging is a complex process involving the cumulative effects of many different genetic pathways in diverse tissues. Efforts to understand the underlying molecular basis of aging are often thwarted by the complexity of the aging process. DNA microarray analysis is well suited to aging research because it allows for simultaneous measurement of gene expression outputs from nearly all genes in the genome in parallel. However, by focusing on changes in expression of genes, most traditional differential analyses neglect the interactions in expression between the genes. For example, the AGEMAP publication described changes in expression levels between young and old mice for the 16 tissues separately [10]. Gene set enrichment analysis (GSEA) was also used to find groups of genes whose expression increased or decreased levels with age. This type of analysis successfully identified many pathways that were previously associated with aging, as well as many novel age-associated pathways. However, there is little overlap between the gene sets found in the AGEMAP paper based on changes in gene expression and the gene sets found in this work based on changes in co-expression interactions. By finding different pathways, both differential expression analysis and co-expression analysis can complement each other to generate a more complete overview of age-related changes.

We used a differential co-expression network approach to show that there are large-scale changes in gene co-expression associated with the aging process. Previous work has shown that there is an increase in the variability in expression levels in old age. Using DNA microarrays, one study showed that expression levels typically show more variability in old versus young, when comparing different samples from either human or rat tissues [32],[33]. Another study used single-cell PCR to show that aging was marked by increased cell-to-cell variation in gene expression in mouse cardiac myoblasts [34]. However, single cell analysis of mRNA levels in a variety of blood cell types did not replicate this finding [35]. These studies all show increased transcriptional instability with age, which is consistent with our finding of a decrease in gene co-expression in old mice.

The loss of correlation in gene expression could be due to several different causes. For instance, transcriptional machinery may degrade with time, such that genes show weaker activation or repression in old tissues compared to young. It could be that there are changes in tissue specificity in old age, such that pairs of genes are co-expressed in specific tissues in young mice but show more general expression across many tissues in old mice. Another possibility is that certain pathways, such as inflammation, could become constitutively induced in old age.

We found two possible mechanisms that could account for loss of gene correlation in old age. The first is that old age may affect the activity of transcription factor NF-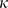
B, such that the direct targets of NF-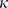
B may show strong co-regulation in young mice but weaker co-expression in old mice. Previous work has also implicated the NF-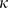
B transcription factor with aging. NF-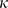
B is involved with inflammation, which increases with age in all tissues [36]. Adler et al. used a combination of differential expression analysis and computational identification of transcription factor targets to identify transcription factors whose targets change expression levels with age [37]. They found that both NF-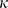
B and AP2 targets increased expression with age. Looking at arrays from three different mouse tissues and six different human tissues they found that their targets were age-induced in the majority of the tissues examined.

NF-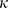
B activity increases with age and controls gene expression through its interaction with the sirtuin protein SIRT6 [38]. A reduction of NF-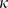
B activity in the skin of old mice caused a reversal of their gene expression aging profile [39]. These results suggest that high or constitutive activity of NF-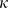
B in old adults could be a molecular mechanism accounting for loss of co-regulation of the NF-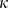
B target genes in old age.

Another possible mechanism for loss of gene co-expression in old age is deterioration of chromatin structure. Histone modifications in chromatin are responsible for both permitting and preventing gene expression [40]. If these histone modifications were to degenerate in old age, chromatin domains would become less well-defined. Genes that are completely repressed and strongly activated in young animals would show either high basal expression or low activated expression in old age. This would result in lower levels of gene co-expression with other genes in the network. In support for a role for chromatin domains in age-regulated changes in transcription, we found that genes that lose correlation with age tend to be clustered together on the chromosome.

The strength of the connections between genes is an important system-wide property of a network that can be used to compare two states. Here, we compare young to old mice, but this approach could be used to compare many other states such as healthy versus disease or wild-type versus mutant. Differential network analysis, is applicable not only to co-expression networks derived from gene expression data, but can also potentially be applied to other types of biological networks, including networks constructed from protein-protein interactions, mutant phenotypes, or from integration of many types of gene interaction experiments. Differential network analysis could potentially be used to compare a network from one species to the network from another. Finally, this approach could be used to evaluate non-biological networks, including changes in social or economic networks over time.

## Materials and Methods

### Data normalization

We downloaded the AGEMAP data from the NCBI Gene Expression Omnibus (accession GSE9909) [10]. The AGEMAP microarray collection contains microarrays for 16 different tissues for five male and five female mice aged both 16 and 24 months. We removed the liver, striatum, and bone marrow samples because they were missing multiple array experiments (i.e., they each had less than four biological repeats for either the males or females in a single age group). For each remaining array, we then calculated the mean correlation coefficient with each of the other four arrays in the same tissue, sex, and age class. For example, for an array taken from the kidney of a 16-month-old male, we calculated the correlation coefficients across all genes for the remaining four male kidney samples. We calculated the mean of those four correlations to determine how well the array agrees with other arrays in the same class. Figure S4 plots the distribution of mean correlation coefficients for each array.

Thereafter, we removed any arrays with a mean correlation coefficient of less than 0.8. In both the young and the old data set there were 2 such arrays. We substituted any missing or removed arrays with a pseudoarray calculated from the mean of the four arrays for the missing arrays' tissue, sex, and age class. The pseudoarray keeps the number of arrays equal for both age groups, ensuring that all tissues are represented equally in the resulting data. The presence of pseudoarrays could potentially bias gene-correlation coefficients toward a higher correlation. However, because there are equal numbers of pseudoarrays in both the young and the old data sets, we found this bias to be acceptable.

Next, we normalized each array by subtracting the mean expression value over all genes for that array. After normalization, we removed all probes that had a low variance and low expression in both the 16-month-old and 24-month-old data sets. We tested the effect of removing low-expressing genes by studying the correlation between probes that match to the same genes. Each array contains 12,273 probes, which map to 8932 unique UniGene IDs [41]. If the mapping is correct, one would expect that the expression of two probes that match to the same UniGene ID would be highly correlated.

Figure S5 plots the expression correlation for each pair of probes that map to the same UniGene ID. By discarding the probes that fall below a set mean (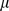) and variance (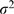), we observed that thresholding reduces the proportion of matched probes with low correlation. By increasing the mean and variance thresholds, we decreased the total number of probes; however, the mean correlation between the matched probes increased. These results indicate that as we removed the genes that have low expression in all arrays, the amount of noise due to nonexpressing genes correspondingly decreased. We ultimately chose cutoffs of 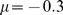 and 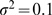, leaving 9104 probes that exceed these thresholds.

### Connectivity and clustering coefficient

If the neighborhood 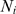 represents the set of directly connected neighbors of gene 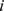, then the connectivity of that node is the number of neighbors in the neighborhood:
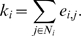
Given a gene's neighborhood (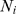) the clustering coefficient is the fraction of links between the nodes in its neighborhood over the total possible number of edges between all genes in the neighborhood:
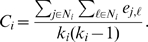
The clustering coefficient can be considered a measure of modularity in the data. Networks that have a tendency toward high clustering coefficients contain many densely connected subgraphs.

### Significance testing: permutation

We used a permutation method to determine the significance for a number of test statistics. For each one, we generated 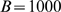 permuted data sets such that each permuted data set contained randomly sampled data from the young and the old data. Because each data set is stratified into sex, mice, and arrays, we sampled from the individual male and female mice separately. For example, for the male mice we pooled the 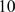 males, 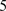 young and 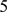 old. We then randomly chose 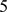 mice from the pool and labeled them young in the randomized data set. The remaining 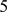 mice were designated as old for the randomized data. For each mouse, all of the arrays and all of the probes went into the same data set. We repeated this procedure for the female mice. We then computed the test statistic on the permuted data and repeated 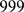 more times.

For each permutation of the data, we recalculated the test statistic and counted the number of times the permuted test statistic exceeded the observed value. For example, let 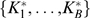 be the set of test statistics generated from the 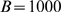 total permutations. Then the *p*-value is calculated as:
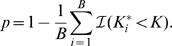
Because of the different stratifications, we end up with 
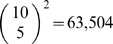
 possible permutations. In each randomized data set the number of young and the number of old mice is not set to be equal. For example the randomized old data set may have 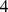 young females and only 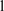 old female. This type of permutation, called non-balanced permutation, has been found to be more accurate than it's balanced counterpart [42].

### Identification of co-expressed clusters from six-month AGEMAP data

We downloaded the AGEMAP data for the 6-month-old mice from the NCBI Gene Expression Omnibus (accession GSE9909) [10]. We normalized the data using the method described for the other AGEMAP data. We found gene clusters by performing a hierarchical clustering of the 6-month data, using a Spearman correlation–based distance metric and average linkage for merging nodes. In average linkage hierarchical clustering, each gene starts as its own cluster. Pairs of clusters are then successively merged according to their average distance. In this case, we used a Spearman correlation based distance metric, 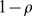, to determine the distance between any two genes. Here 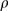 is the Spearman correlation between two genes calculated across all available experiments in the 6-month data set. To determine the distance between two clusters for merging, we used the average distance between all of the genes in the two clusters. Clustering of this sort provides a hierarchical tree of clusters. By cutting the tree at an average distance of 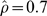, we obtained distinct clusters. We discarded any clusters containing fewer than five genes.

### Calculating cluster overlap with GO, KEGG, and INTERPRO categories

We obtained GO, KEGG and Interpro categories from the DAVID database [43],[44]. For GO categories we looked at gene groupings based on GO molecular function, associated cellular component, and biological process. We discarded any categories with fewer than 5 or more than 200 genes. We determined overlap between a cluster and a functional category using the hypergeometric distribution 
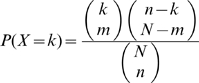
. Here 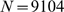 is the number of genes, 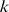 is the number of genes that are both in the cluster and the functional category, 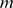 is the size of the cluster and 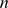 is the size of the functional categories.

### Testing uniform correlation loss

For the node-deletion simulation, we randomly selected nodes in the 16-month-old network and deleted all edges leading out of those nodes. We iterated the edge-selection and deletion process until approximately 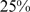 of the edges were deleted, i.e., when the simulated network has the same number of edges as the 24-month-old network. Because it is not possible to exactly match the number of edges in the simulated network to the number of edges in the 24-month network, we allowed 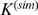 to be within 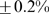 of 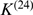, where 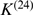 and 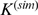 are the total number of nodes in the 24-month and simulated networks, respectively.

We repeated the simulation 100 times, and each time we drew the boundaries of the simulated networks on the scatter plot of the clustering coefficient versus the connectivity. We drew the boundaries by dividing the 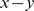 plot into a 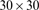 grid and binning the data points for each gene. For each simulation, we then drew a boundary around the outermost edge of the bins that contained at least one data point, such that all bins outside of the drawn boundary contained zero data points.

### Testing modular correlation loss

We defined gene clusters using a range of cluster thresholds, and removed the clusters from the network simulating modular co-expression loss. Clusters were assigned using average linkage hierarchical clustering, with a distance metric of 1-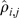, where 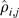 is the Spearman correlation between genes 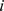 and 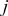. Distinct clusters are formed by cutting the tree at a particular height 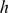. For a given 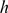, all genes in the resulting cluster have an average distance from one another of at least 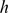. For example, if 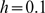, all the clusters that result from cutting the tree at 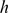 have a mean distance of 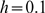 (corresponding to a mean correlation of 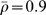). In this way, 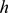 sets the stringency for inclusion into a cluster: small values of 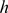 result in clusters of genes that show higher levels of co-expression.

### Finding conserved transcription factor binding data in the mouse genome

We obtained predicted transcription start sites for all human RefSeq genes from the University of California, Santa Cruz (UCSC) human genome assembly (hg17). We then downloaded from the Transfac Matrix Database all of the conserved transcription factor binding sites (TFBs) found by Hinrichs et al. to be at 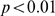. The database contains 258 transcription factors conserved in human, mouse, and rat at this threshold [45]. We then located all of the conserved TFBs within 5000 bp of the transcription start site of all RefSeq genes. Thus, we obtained a list of conserved TFBs within 5000 bp of a known human gene. To map these results to the mouse genome, we used the chained alignment of the mouse genome (*mm9*) to the human genome (hg17) supplied by the UCSC genome database [46]. For every RefSeq gene in *mm9*, we assigned a TFB if the site in the human genome appeared in a conserved region within 5000 bp of the mouse gene.

### Moving window approach for chromosomal clustering

To search for chromosomally-clustered genes, we first defined the correlation loss score of a given gene to be the connectivity of that gene within the difference network. The connectivity here is defined by the number of neighbors a gene 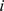 has with 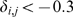 for 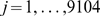, where 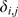 is the edge weight of the difference network between two genes. We then mapped probes to the mouse genome using the *mm9* assembly from the UCSC Genome Browser [47]. When more than one probe mapped to the same location, we averaged the connectivities of those probes.

We used a moving window approach to define gene clusters as follows. First, we identified all genes whose connectivity 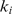 was greater than or equal to a threshold 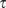. Then, we scanned a window across the genome and counted the number of windows containing more than one gene (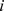) with 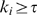. We defined a cluster as a window containing more than one gene that met or exceeded this threshold.

For a given window size and threshold, we calculated the number of gene clusters on the chromosome, then permuted the data to determine whether the rate of clustering exceeded the rate expected by chance. For every chromosome, we permuted the genes' locations and recalculated the number of gene clusters. We defined the two sided *p*-value as 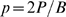, where 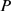 is the number of gene clusters in the permuted set that exceeded the original number of clusters, and where 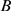 is the number of permutations.

## Supporting Information

Figure S1 The difference in the number of clusters using *d^+^* and *d^−^* for the 1,000 permutations. None of the permuted differences in cluster number (absolute) is larger than the real difference in cluster numbers.
(0.26 MB EPS)

Figure S2 The clustering coefficient (*cc*) versus the connectivity (*k*) for young and old mice as contrasted with the modular deletion simulation. Each dot represents a probe in either the 16-month-old (*blue*) and 24-month-old (*red*) networks. All of the probes with at least one neighbor are plotted. The distributions from the cluster-deletion simulations are shown in gray. For each of the four panels, a different height parameter was chosen for the clustering.
(10.09 MB TIF)

Figure S3 Significance of chromosomal clustering. (A) A histogram of the number of clusters found in the permuted data (window size *l = 80* kb and threshold *τ = 6*). Of the 1,000 permutations, only two surpassed the real value of 44 clusters. (B) The percent of permuted chromosomal clusters that surpass the true value for a variety of window sizes and thresholds. The *blue* line corresponds to *τ = 2*, the *green* line corresponds to *τ = 4*, and the *red* line corresponds to *τ = 6*. The dotted line represents the *2.5th* percentile corresponding to *p<0.05*.
(0.46 MB EPS)

Figure S4 The distributions for each array's correlation with other arrays in the same sex and tissue class for each age group. If an array was missing due to experimental error, the correlation was plotted as zero. Using a cutoff of *ρ* = 0.8, we excluded two arrays from both the 16-month-old and 24-month-old data.
(0.46 MB EPS)

Figure S5 Removal of genes with low expression mean and variance. (A) For a variety of mean (*μ*) cutoffs,we calculated the correlations between all pairs of probes that map to the same UniGene ID. The white bars indicate the correlation between matched probe pairs for all possible pairs. The red bars represent the correlations of the remaining probes after thresholding. (B) Plot of the fraction of matched probe pairs with *ρ*>*0.5* after imposing a variety of mean (*μ*) and variance (*σ^2^*) thresholds. As the thresholds increase, the number of probes that pass the thresholds (*n*) decreases.
(0.39 MB EPS)

Table S1 List of chromosome clusters by chromosome.
(0.03 MB PDF)
