# Supplementary figures and images for: Aging Mice Show a Decreasing Correlation of Gene Expression within Genetic Modules
Source: PLoS Genet. 2009 Dec 18;5(12):e1000776. doi: 10.1371/journal.pgen.1000776 (PMC2788246; doi:10.1371/journal.pgen.1000776)

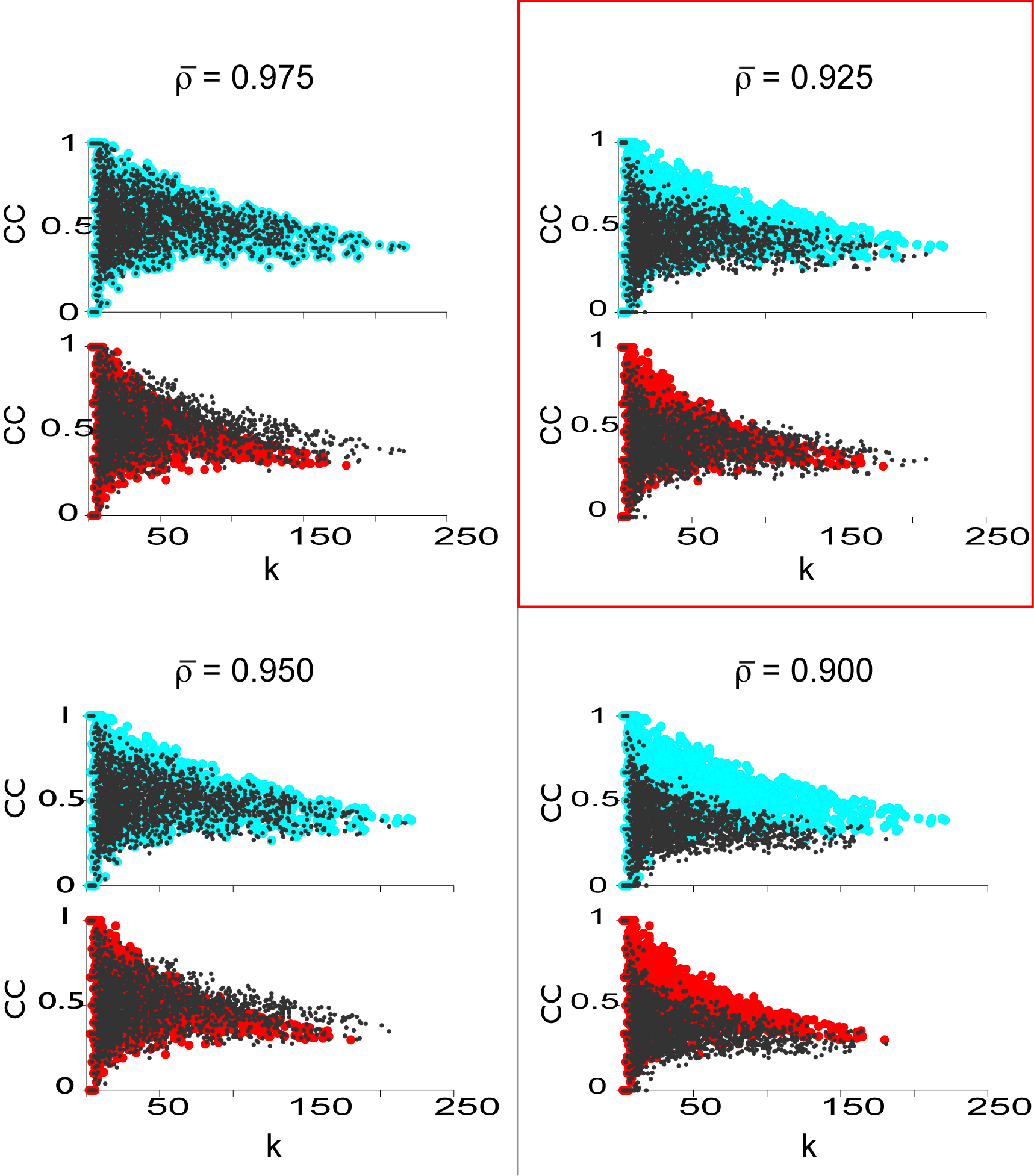

Supplement: Figure S2 — The clustering coefficient (cc) versus the connectivity (k) for young and old mice as contrasted with the modular deletion simulation. Each dot represents a probe in either the 16-month-old (blue) and 24-month-old (red) networks. All of the probes with at least one neighbor are plotted. The distributions from the cluster-deletion simulations are shown in gray. For each of the four panels, a different height parameter was chosen for the clustering. (10.09 MB TIF) [file pgen.1000776.s002.tif]
