# Supplementary material for: Aging Mice Show a Decreasing Correlation of Gene Expression within Genetic Modules
Source: PLoS Genet. 2009 Dec 18;5(12):e1000776. doi: 10.1371/journal.pgen.1000776 (PMC2788246; doi:10.1371/journal.pgen.1000776)
Supplement: Table S1 — List of chromosome clusters by chromosome. (0.03 MB PDF) [file pgen.1000776.s006.pdf]

Supplementary Table 1: **List of Chromosome Clusters by Chromosome**

**Cluster ID**	**Chromsome number**	**Genbank ID**	**Locus Link ID**	**Gene Position (TSS)**
Cluster 1	Chromosome 1	BG082513<br>BQ550188	245860<br>20872	75188497<br>75207446
Cluster 2	Chromosome 1	BG086586<br>BG085359	12847<br>19298	174044467<br>174056902
Cluster 3	Chromosome 2	BG073109<br>BG064709	277396<br>20823	69660529<br>69699684
Cluster 4	Chromosome 2	BQ550512<br>BG074386	140629<br>77006	130455722<br>130490381
Cluster 5	Chromosome 2	BG084735<br>BG074245	99237<br>54711	153027872<br>153067094
Cluster 6	Chromosome 2	BG077290<br>BG074423	20878<br>67337	172196025<br>172196502
Cluster 7	Chromosome 2	BG087188<br>BG076209	228960<br>228961	173902551<br>173935865
Cluster 8	Chromosome 3	BG085485<br>BG078740	53814<br>78523	94236335<br>94247257
Cluster 9	Chromosome 3	BG075921<br>BG084805	320988<br>319916	94778657<br>94801272
Cluster 10	Chromosome 3	BG084805<br>BG086311	319916<br>19185	94801272<br>94846467
Cluster 11	Chromosome 3	BG088653<br>BG073154	57912<br>171388	95032873<br>95055024
Cluster 12	Chromosome 4	BG069856<br>BG071074	51813<br>77593	21654876<br>21703416
Cluster 13	Chromosome 4	BG071285<br>BG077295	72181<br>66576	115726481<br>115747675
Cluster 14	Chromosome 4	BG077295<br>BG075033	66576<br>230654	115747675<br>115748069
Cluster 15	Chromosome 4	BG082743<br>BG078275	69902<br>230866	138908213<br>138908507
Cluster 16	Chromosome 4	BG070654<br>BG071722	381569<br>194225	143405982<br>143436399
Cluster 17	Chromosome 6	BG066677	319801	127059554

		AW547625	12444	127101066
Cluster 18	Chromosome 7	AW552582<br>BG064628	54006<br>98845	148524660<br>148551009
Cluster 19	Chromosome 8	BG069650<br>C81248	102226<br>102182	80041806<br>80073295
Cluster 20	Chromosome 8	BG083923<br>BG085360	320394<br>68051	108375908<br>108384533
Cluster 21	Chromosome 9	BG087957<br>BG064919	68682<br>16201	21142295<br>21192143
Cluster 22	Chromosome 9	BG076387<br>BQ550191	19340<br>66962	21722565<br>21760198
Cluster 23	Chromosome 9	BG063933<br>BQ550683	13478<br>15288	44134927<br>44152259
Cluster 24	Chromosome 9	BG087927<br>BG071194	72278<br>11891	72863464<br>72912049
Cluster 25	Chromosome 10	BG063817<br>BG076890	67895<br>20224	61111368<br>61143068
Cluster 26	Chromosome 10	BG077044<br>BG073436	56351<br>11947	127496037<br>127520362
Cluster 27	Chromosome 10	BG073436<br>BG066189	11947<br>116848	127520362<br>127558646
Cluster 28	Chromosome 10	BG064664<br>BG064819	23943<br>18813	127962915<br>128002990
Cluster 29	Chromosome 11	BG085864<br>BG066516	114679<br>29856	3414704<br>3439242
Cluster 30	Chromosome 11	BG083639<br>BG063430	24070<br>13681	69476144<br>69485817
Cluster 31	Chromosome 11	BG088662<br>BG072388	103737<br>71770	83112479<br>83116198
Cluster 32	Chromosome 11	BG064529<br>AU042878	21428<br>19183	100948638<br>100956715
Cluster 33	Chromosome 11	BG085903<br>BG063439	237988<br>71679	115243229<br>115281233
Cluster 34	Chromosome 14	BG086188<br>BG071033	19270<br>Inf	12386066<br>12386066
Cluster 35	Chromosome 14	BG073963<br>BG070657	219158<br>268759	70553598<br>70559278
Cluster 36	Chromosome 15	BG085919<br>BG086876	16469<br>23936	74539752<br>74583409

Cluster 37	Chromosome 15	BG073090<br>BG064620	29870<br>72026	85690405<br>85724636
Cluster 38	Chromosome 16	BG080326<br>BG064304	74112<br>12469	87455300<br>87496114
Cluster 39	Chromosome 17	BG078866<br>BG070867	56424<br>68347	25970306<br>26012461
Cluster 40	Chromosome 17	BG070867<br>BQ550575	68347<br>224624	26012461<br>26056647
Cluster 41	Chromosome 17	BG079374<br>BG063876	50782<br>66978	26344786<br>26389854
Cluster 42	Chromosome 19	C78065<br>BG077817	83673<br>53319	8797976<br>8831592
Cluster 43	Chromosome 19	BG064032<br>BG087370	225896<br>14376	8946082<br>8972600
Cluster 44	Chromosome 19	BG070958<br>BG066686	56350<br>94279	46647575<br>46647854
